# Genomic insights into activated antimicrobial resistance of *in situ* hospital-wastewater biofilm

**DOI:** 10.1016/j.bioflm.2026.100377

**Published:** 2026-06-23

**Authors:** Yusuke Ota, Yoko Nukui, Yoshiaki Gu, Ryoichi Saito

**Affiliations:** a Department of Molecular Microbiology and Immunology, Graduate School of Medical and Dental Sciences, Institute of Science Tokyo, Tokyo, Japan; b Department of Infection Control and Laboratory Medicine, Kyoto Prefectural University of Medicine, Kyoto, Japan; c Department of Infectious Diseases, Graduate School of Medicine and Dental Sciences, Institute of Science Tokyo, Tokyo, Japan

**Keywords:** Hospital wastewater, Antimicrobial resistance, Biofilm, *bla*_IMP-1_, Tn*3* family transposon

## Abstract

Antimicrobial resistance (AMR), particularly among carbapenemase-producing organisms, poses a major global health threat. Although hospital wastewater is considered an AMR hotspot, its functional contribution to resistance dynamics remains poorly defined. We developed *in situ* biofilms in hospital wastewater and applied integrated metagenomic, metatranscriptomic, and culture-based analyses to characterize community structure and gene expression. Biofilms exhibited greater biomass and higher contamination with extended-spectrum β-lactamase-producing *Escherichia coli* than planktonic wastewater. Biofilms were enriched in surface-adapted *Flavobacteriaceae* species and a broader array of carbapenemase genes, whereas wastewater showed higher abundance of gut-associated *Bacteroidaceae* species and virulence factors. Mobile genetic elements linked multiple AMR genes and showed increased expression in biofilms, including *bla*_IMP_ family carbapenemases. Culture confirmed *bla*_IMP-1_ in four biofilm isolates and one wastewater isolate. These findings indicate that hospital-wastewater biofilms can serve as important reservoirs that promote the persistence and potential dissemination of clinically relevant carbapenem resistance.

## 1 Introduction

Antimicrobial-resistant pathogens have become a critical global health threat, as they increasingly compromise the effectiveness of infectious disease treatments. Global burden estimates for antimicrobial resistance (AMR) in 2021 indicate 4.71 million AMR pathogen-related deaths worldwide, with projections suggesting that related mortality will reach approximately 8.22 million by 2050 [1]. Among antimicrobial-resistant pathogens, carbapenemase-producing organisms are particularly concerning because they exhibit a multidrug resistance phenotype and are associated with worse clinical outcomes, including high mortality, long durations of antibiotic therapy, and prolonged hospital stays, compared to those without this phenotype [2]. Clinically important AMR spreads through mobile genetic elements, including transposons and integrons, highlighting the need for comprehensive AMR monitoring to formulate effective control strategies [3].

A One Health approach is essential for combating AMR because antimicrobial-resistant organisms circulate across humans, animals, and various environments, requiring coordinated cross-sectoral action [4]. Hospital wastewater represents a critical environmental reservoir and transmission interface for AMR, reflecting selective pressures within healthcare settings and contributing to the dissemination of antimicrobial-resistant pathogens beyond clinical boundaries [5]. Consistently, hospital wastewater contains diverse antimicrobial-resistant organisms, including carbapenemase producers, as well as selective substances such as antimicrobial residues, chemical agents, and heavy metals that promote the persistence and dissemination of AMR [5,6]. Moreover, emerging forms of AMR have frequently been detected in environmental sources prior to their recognition in clinical settings, underscoring the utility of hospital wastewater for not only monitoring the current circulation of AMR but also identifying future epidemic trends [7].

Biofilms readily form in aquatic environments such as hospital wastewater, consisting of surface-attached, matrix-embedded microbial communities. Within biofilms, AMR is effectively sustained through impaired antibiotic penetration, reduced bacterial metabolic activity, and matrix-mediated physiological states that diminish antimicrobial effectiveness [8,9]. Furthermore, biofilms facilitate active horizontal gene transfer, mediated by mobile genetic elements, thereby accelerating the diversification and spread of AMR within microbial communities [10]. Environmental studies have demonstrated a marked increase in the uptake of extracellular DNA within biofilms, highlighting their enhanced capacity for horizontal gene acquisition [11]. Accordingly, a comprehensive understanding of environmental AMR requires not only conventional analyses of AMR abundance in planktonic communities but also evaluation of highly active genes, including AMR determinants and mobile genetic elements, within biofilms.

Plastics in aquatic environments act as active carriers and enrichment niches for antimicrobial-resistant bacteria and AMR genes, and the biofilms that form on their surfaces exhibit AMR profiles distinct from those in the surrounding water [12,13]. A recent study has shown that hospital wastewater contains substantial amounts of plastic debris, which can act as environmental vectors that facilitate the spread and evolution of AMR [14]. In this study, *in situ* biofilms were developed on plastic slides in hospital wastewater, and the distribution and expression levels of AMR genes were comprehensively characterized using metagenomic and metatranscriptomic analyses. Additionally, antimicrobial-resistant bacteria that exhibited high transcriptional levels were isolated through culture-based methods, enabling comparison of their genetic and phenotypic properties. This study provides insights into the mechanisms underlying the emergence and dissemination of biofilm-associated AMR, with important implications for understanding and mitigating the growing problem of AMR in the environment.

## 2 Methods

### 2.1 Biofilm development and sample collection

**Fig. 1:**
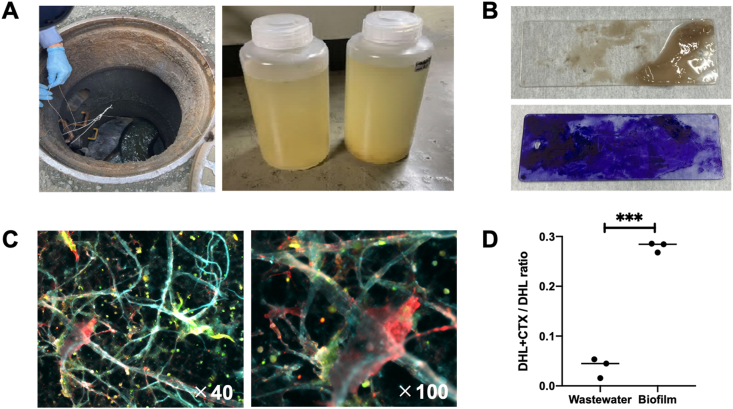
Characteristics of *in situ* biofilm and hospital wastewater samples. (**a**) Placement of plastic slides in hospital wastewater for *in situ* biofilm formation and collection of wastewater sample. (**b**) *In situ* biofilm samples before and after crystal-violet staining. (**c**) Fluorescent staining of *in situ* biofilm structure: green, red, and blue represent nucleic acids, proteins, and polysaccharides, respectively. (**d**) Comparison of contamination levels based on antimicrobial resistance between hospital wastewater and *in situ* biofilm. Statistical analysis using unpaired *t*-test; ∗∗∗p < 0.0001. DHL: deoxycholate hydrogen sulfide lactose, CTX: cefotaxime. (For interpretation of the references to color in this figure legend, the reader is referred to the Web version of this article.)

*In situ* biofilms were established in hospital wastewater following a previously described method [15]. Polystyrene slides (25 mm × 75 mm; thickness, 1.0 mm) (Kenis, Osaka, Japan) were placed in wastewater at the Institute of Science Tokyo Hospital, which is a teaching hospital with more than 800 beds, on October 7, 2024. On October 15, 2024, after 8 d of exposure to hospital wastewater, the polystyrene slides with *in situ* biofilms were retrieved, and the wastewater was collected in a sterile bottle (Fig. 1a). These samples were processed within 1 h of collection. The hospital wastewater was concentrated 10-fold by centrifugation. The slides with biofilms were rinsed with sterile phosphate-buffered saline (PBS). These slides were used as follows: one slide each for crystal-violet staining, fluorescent staining, bacterial isolation, and shotgun metagenomic sequencing analysis and three independent slides for evaluating the proportion of extended-spectrum β-lactamase (ESBL)-producing *Escherichia coli* and shotgun metatranscriptomic sequencing analysis. Slides for crystal-violet staining were stored in PBS, while slides for fluorescent staining were fixed in 3.7% formaldehyde for 1 h, washed with PBS, and air-dried. For DNA and RNA extraction, the biofilm was scraped off the slides, preserved in PBS for bacterial culture, and stored in DNA/RNA Shield (Zymo Research, Irvine, CA, USA).

### 2.2 Staining of biofilm components

For crystal-violet staining, the slides with biofilms were stained with 0.1% crystal violet (Muto Chemical, Tokyo, Japan) for 30 min and then washed with PBS. For fluorescence staining, nucleic acid was stained with cell-permeant SYTO 9 (Thermo Fisher Scientific, Waltham, MA, USA), proteins were stained with FilmTracer™ SYPRO™ Ruby Biofilm Matrix Stain (Thermo Fisher Scientific), and polysaccharides were stained with Concanavalin A conjugated to tetramethylrhodamine (Thermo Fisher Scientific). Fluorescent-stained slides were observed using a BZ-X810 microscope (KEYENCE, Osaka, Japan).

### 2.3 Bacterial isolation

To assess AMR contamination in environmental samples, we followed the World Health Organization Tricycle protocol and determined the prevalence of ESBL-producing *E. coli* [16]. Biofilm and wastewater samples were inoculated on deoxycholate hydrogen sulfide lactose agar plates containing 4 μg/mL cefotaxime and on plates without the antibiotic (control). Five presumptive *E. coli* colonies from each plate were selected and tested for indole production. ESBL production by these *E. coli* colonies was confirmed using the double-disc synergy test based on the Clinical and Laboratory Standards Institute (CLSI) M100-S32 guideline. The proportion of ESBL-producing *E. coli* among all *E. coli* isolates was compared across samples, and statistical significance was evaluated using an unpaired *t*-test based on biological triplicates. Next, the biofilm and wastewater samples were plated on CHROMagar selective agar (Kanto Chemical, Tokyo, Japan) for the isolation of methicillin-resistant *Staphylococcus aureus*, vancomycin-resistant enterococci, ESBL producers, carbapenemase producers, multidrug-resistant *Pseudomonas aeruginosa*, and multidrug-resistant *Acinetobacter* species. Plates were incubated under ambient air conditions at 37°C overnight. Colonies with distinct morphologies were identified through 16S rRNA gene sequencing based on the closest sequence match [17]. Selected colonies were tested for the presence of carbapenemase-encoding genes (i.e., *bla*_IMP_, *bla*_VIM_, *bla*_KPC_, *bla*_GES_, *bla*_OXA-48-like_, and *bla*_NDM_) following standard polymerase chain reaction (PCR) protocols, as previously described [18,19].

### 2.4 Shotgun metagenomic sequencing analysis

Genomic DNA was extracted from wastewater and biofilm samples using the QIAamp PowerFecal Pro DNA Kit (QIAGEN, Hilden, Germany). Libraries were prepared using the QIAseq FX DNA Library Kit (QIAGEN) and the MGIEasy Universal Library Conversion Kit (MGI Tech, Shenzhen, China), and sequencing was performed on the DNBSEQ-G400 platform. Adapter trimming and low-quality read removal were performed using bbduk.sh (version 39.01; https://sourceforge.net/projects/bbmap/). Reads were mapped to a masked human reference genome using bbmap.sh (version 39.01; https://sourceforge.net/projects/bbmap/), and those aligning to the human genome were removed to eliminate human genome contamination. *De novo* assembly was performed using SPAdes (version 3.15.5) [20]. Contigs shorter than 200 bp were removed using seqkit (version 2.6.1) [21]. Binning was performed using MetaBAT2 (version 2.15) [22] and MaxBin2 (version 2.2.7) [23] to construct MAGs. The MAGs obtained through binning process were merged using MAGScoT (version 1.0.0) [24]. The completeness and contamination rate of MAGs were assessed using CheckM (version 1.1.3) [25]. Taxonomy of MAGs was assigned using GTDB-Tk (version 2.3.2) [26]. AMRFinderPlus (version 4.0.15) [27] was used to predict AMR genes, metal-resistance genes (MRGs), biocide-resistance genes (BRGs), and heat-resistance genes (HRGs). Plasmid-derived sequences were identified using Platon (version 1.6) [28]. The insertion sequences (ISs) from ISfinder [29] and the major integron sequences *intI1* (AB709942), *intI2* (JX469830), and *intI3* (EF467661) [30] were retrieved from TnCentral [31] and the NCBI database [32], respectively (accessed on January 22, 2025), and these sequences were detected in shotgun genomic data using BLAST (version 2.15.0) [33]. A gene co-occurrence network was generated using Cytoscape (version 3.10.3) [34].

### 2.5 Shotgun metatranscriptomic sequencing analysis

Total RNA was extracted from hospital wastewater and biofilm samples, each analyzed in biological triplicate with independently prepared biofilm samples, using the RNeasy PowerMicrobiome Kit (QIAGEN). Ribosomal RNA was depleted using the QIAseq FastSelect–5S/16S/23S Kit (QIAGEN). Library preparation was performed using the QIAseq Stranded RNA Library Kit (QIAGEN) and the MGIEasy Universal Library Conversion Kit (MGI Tech), and the DNBSEQ-G400 platform (MGI Tech) was used for sequencing. Quality filtering was performed using Fastp (version 0.19.5) [35]. The obtained reads were mapped to the reference sequences of AMRFinderPlus [27], PLSDB [36], ISfinder [29], and major integrons [30] using HISAT2 (version 2.1.0) [37]. Primary mapped reads were extracted using Samtools (version 1.10) [38]. Read counts for each gene were obtained using featureCounts (version 2.0.3) [39] and CoverM (version 0.6.1) [40]. Gene expression values were normalized as transcripts per million (TPM) to account for differences in gene length and sequencing depth. Differential expression analysis between wastewater and biofilm samples was performed using an unpaired *t*-test for each gene to compare group means. To correct for multiple testing, false discovery rates (FDRs) were subsequently calculated using the “edgeR” package in R [41].

### 2.6 Whole-genome sequencing analysis

Bacterial DNA was extracted from colonies of each isolate using the NucleoBond HMW DNA Kit (Macherey-Nagel, Düren, Germany). Library preparation was performed for Illumina short reads using the Nextera DNA Flex Library Preparation Kit (Illumina, San Diego, California, USA). Whole-genome sequencing using paired-end reads was performed on the Illumina MiniSeq platform. Long-read DNA libraries were prepared using the Ligation Sequencing Kit and sequenced using the Nanopore MinION sequencer and R10.4.1 flow cells (Oxford Nanopore Technologies, Oxford, UK). Raw short reads were processed for filtering and trimming with Fastp (version 0.19.5) [35], while long reads were quality-filtered using Porechop (version 0.2.4) [42] and Filtlong (version 0.2.1) [43]. The filtered reads were assembled into hybrid genomes *de novo* using Unicycler (version 0.5.0) [44]. Sequencing depth of bacterial isolate genomes was assessed using seqkit (version 2.8.0) [21] and genome completeness was evaluated using BUSCO (version 6.0.0) [45]. Bacterial species were determined using GTDB-Tk Classify (version 1.7.0) [26] based on average nucleotide identity values. Sequence type was determined by multi-locus sequence typing [46]. Plasmid incompatibility type, AMR genes, and ISs were identified using PlasmidFinder (version 2.1) [47], AMRFinderPlus [27], and ISfinder [29], respectively. EasyFig (version 2.2.2) [48] was used to align assembled reads containing carbapenemase genes. Following annotation with Prokka (version 1.13) [49], pan-genome analysis was performed using Roary (version 3.13.0) [50], incorporating complete genome sequences of 20 *Citrobacter* species strains from previous studies [51,52]. A maximum-likelihood tree was built using IQ-TREE (version 2.3.0) [53] and visualized with iTOL (version 7) [54].

### 2.7 Antimicrobial susceptibility testing

The broth microdilution method was performed using the MicroScan Neg Combo EN5J (*Enterobacterales* and *Aeromonas* sp.) and NF2J (*Pseudomonas* sp.) panels (Beckman Coulter, Brea, CA, USA). Minimum inhibitory concentrations (MICs) of antibiotics for the bacterial isolates were determined using the MicroScan autoSCAN-4 system (Beckman Coulter). The breakpoints for antimicrobial susceptibility testing were interpreted according to the CLSI documents M100-Ed32 and M45-Ed3.

## 3 Results

### 3.1 Establishment and characterization of *in situ* biofilm in hospital wastewater

*In situ* biofilm was successfully established on polystyrene slides after 8 d of exposure to hospital wastewater (Fig. 1a). Crystal-violet staining confirmed substantial biofilm biomass on the slide surface (Fig. 1b). Fluorescence microscopy revealed a heterogeneous biofilm architecture with an extensive extracellular polymeric substance (EPS) matrix, characterized by filamentous structures and interconnected patterns that indicate a complex three-dimensional (3D) organization (Fig. 1c). The EPS contained multiple structural components, including nucleic acids, proteins, and polysaccharides, forming an intricate network that provided mechanical stability. Comparative analysis of AMR revealed the presence of ESBL-producing *E. coli* in both biofilm and hospital wastewater samples, with the mean ratio being markedly higher in biofilm samples (0.279) than in hospital wastewater samples (0.038) (Fig. 1d).

### 3.2 Taxonomic structure and functional gene distribution

**Fig. 2:**
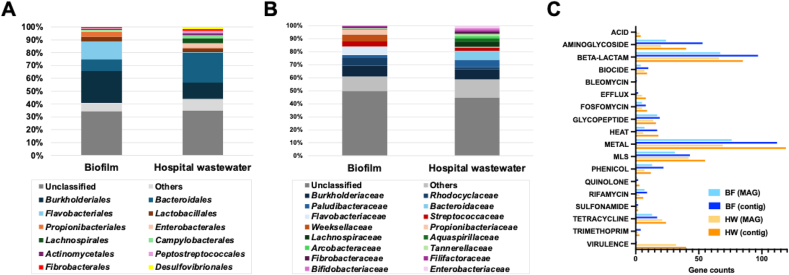
Taxonomic and functional gene profiles of *in situ* biofilm and hospital wastewater based on shotgun metagenomic data. Relative abundance of bacterial taxa at the order (**a**) and family (**b**) levels in *in situ* biofilm and hospital wastewater samples. (**c**) Distribution of genes associated with resistance to antibiotics, biocides, acids, heat, and metals, as well as virulence, shown at the metagenome-assembled genome (MAG) and contig levels for biofilm (BF) and hospital wastewater (HW). MLS: macrolide-lincosamide-streptogramin.

Shotgun metagenomic sequencing revealed distinct taxonomic profiles between biofilm and hospital wastewater samples (Supplementary Table S1–S3). At the order level, *Bacteroidales* and *Burkholderiales* were dominant in hospital wastewater, whereas biofilms exhibited higher relative abundance of *Burkholderiales* and *Flavobacteriales* (Fig. 2a). At the family level, *Burkholderiaceae* was predominant in both the hospital wastewater and biofilm samples. Additionally, *Bacteroidaceae* and *Flavobacteriaceae* exhibited high relative abundance in hospital wastewater and biofilm samples, respectively. Functional annotation of metagenome-assembled genomes (MAGs) and contigs indicated widespread occurrence of AMR genes, MRGs, BRGs, and HRGs in the biofilm and hospital wastewater samples (Fig. 2c). The AMR genes conferring resistance to β-lactams, aminoglycosides, and macrolide-lincosamide-streptogramin were highly expressed in both sample types. Carbapenemase genes identified in biofilm samples included *bla*_GES-5_, *bla*_IMP-1_, *bla*_IMP-11_, *bla*_KPC-2_, *bla*_OXA-23_, *bla*_OXA-58_, and *bla*_NDM-1_, whereas hospital wastewater contained *bla*_GES-5_ and *bla*_KPC-2_ (Supplementary Tables S4 and S5). Virulence-associated genes were predominantly detected in hospital wastewater, including adhesion-related genes (*eilA*, *fdeC*, *lpfA*-O113, *pap*, and *sfa*), toxin and effector-related genes (*cif*, *cnf1*, *esp*, *hlyA*, *nleB*, *tir*, and *vat*), iron acquisition system-related genes (*iro*, *iuc*, and *ybt*), and other virulence factor genes (*cvaC*, *iss*, *mchF*, *senB*, *sinH*, and *sslE*). By contrast, only *iss* was detected in the biofilm sample.

### 3.3 Co-occurrence structures of genomic components

**Fig. 3:**
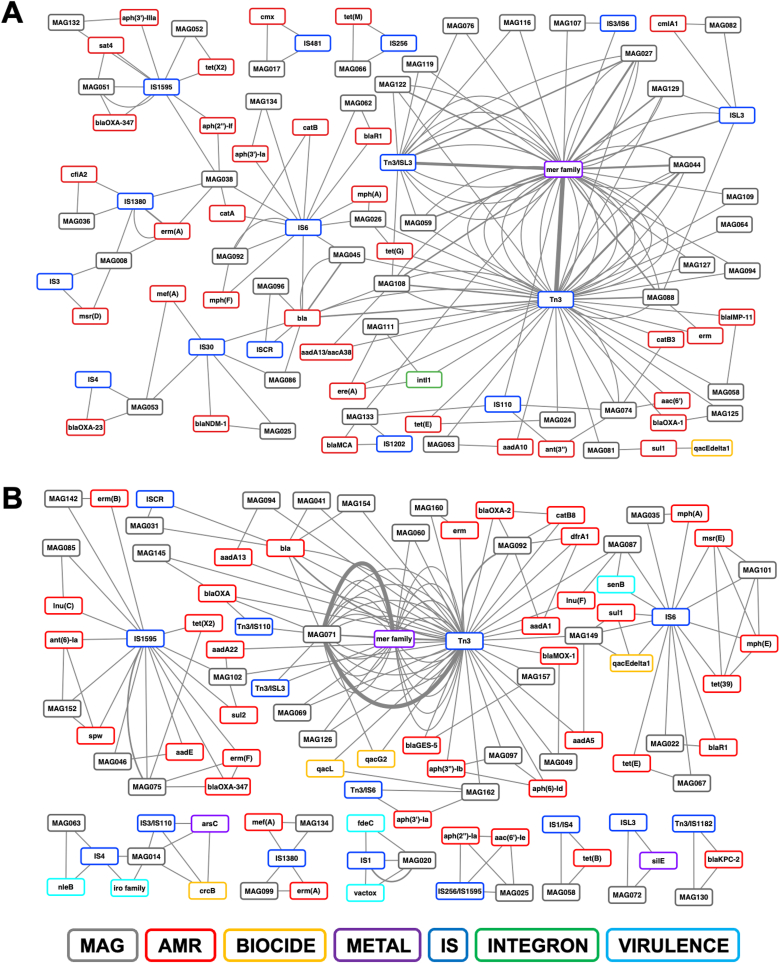
Network analysis of metagenome-assembled genomes, functional genes, and mobile genetic elements based on shotgun metagenomic data. Networks of metagenome-assembled genomes (MAGs) and genes related to antimicrobial resistance (AMR), biocide and metal resistance, insertion sequences (ISs), integron, and virulence are depicted for biofilm (**a**) and hospital wastewater (**b**) samples. Nodes represent individual sequences and edges indicate co-occurrence within the same MAG or contig.

Network diagrams were constructed to represent co-occurrence relationships among MAGs and functional genes. These networks exhibit centralized architecture dominated by a few highly connected hubs, notably Tn*3* family transposons and ISs, in both *in situ* biofilm (Fig. 3a) and wastewater (Fig. 3b) samples. These hubs are strongly associated with multiple AMR genes and MAGs, forming dense clusters. Among carbapenemase genes detected in biofilm, *bla*_IMP-11_, *bla*_OXA-23_, and *bla*_NDM-1_ exhibited co-occurrence with MAGs and mobile genetic elements: *bla*_IMP-11_ with *Bacteroidales* MAG058 and Tn*3*; *bla*_OXA-23_ with *Bacteroidales* MAG053 and IS*4*; *bla*_NDM-1_ with *Burkholderiales* MAG025 and IS*30* (Supplementary Table S2). In the wastewater sample, *bla*_KPC-2_ was associated with *Psittacicellaceae* MAG130 and Tn*3*/IS*1182*, whereas *bla*_GES-5_ was linked to unclassified MAG157 and Tn*3* (Supplementary Table S3). We also evaluated the co-occurrence relationships among plasmid-derived contigs and functional genes (Supplementary Fig. S1). Tn*3* served as a central hub, while IS*6* and IS*1595* exhibited multiple connections to plasmid-derived contigs and AMR genes. *bla*_IMP-11_ was associated with Tn*3* and *bla*_NDM-1_ with IS*30* in the biofilm samples, whereas *bla*_KPC-2_ was linked to Tn*3*/IS*1182* in the hospital wastewater samples.

### 3.4 Comparative analysis of gene expression profiles

**Fig. 4:**
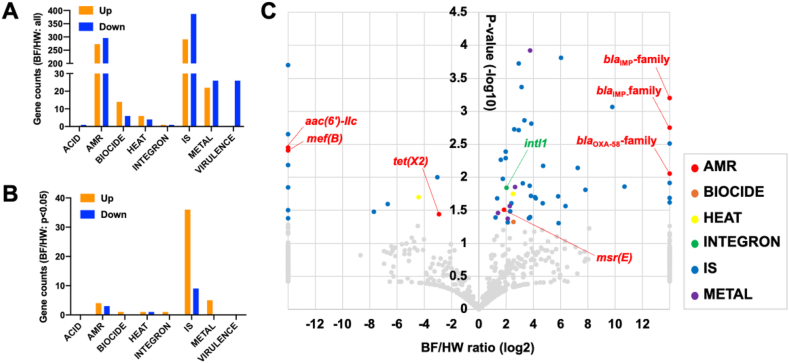
Differential gene expression profiles between biofilm and hospital wastewater based on metatranscriptomic data. (**a**) Number of genes with higher (Up) or lower (Down) expression in biofilm (BF) compared to hospital wastewater (HW) across all genes. (**b**) Number of genes with significantly different expression (p < 0.05) between BF and HW. (**c**) Volcano plot showing genes with significant expression differences (p < 0.05) between BF and HW, where colors describe functional categories. (For interpretation of the references to color in this figure legend, the reader is referred to the Web version of this article.)

Metatranscriptomic comparison between the biofilm and hospital wastewater samples revealed clear differences in gene expression profiles (Supplementary Table S6). Across all detected genes, numerous BRGs and HRGs showed higher expression in biofilm, whereas AMR genes, IS, MRGs, and virulence genes exhibited higher expression in the hospital wastewater (Fig. 4a). When statistical significance was applied (p < 0.05), a distinct subset of genes was identified as significantly upregulated or downregulated in biofilms relative to that in hospital wastewater (Fig. 4b). The volcano plot further demonstrated that each gene exhibiting significant differences in expression was distributed across diverse functional categories (Fig. 4c). *aac(6’)-IIc*, *mef(B)*, and *tet(X2)* were highly expressed in the wastewater, whereas *bla*_IMP_ family, *bla*_OXA-58_ family, and *msr(E)* expression was markedly upregulated in the biofilm, with the *bla*_IMP_ family showing a notable difference. Additionally, the expression of the *bla*_IMP_ family and *bla*_OXA-58_-family was significantly upregulated in the *in situ* biofilm according to the FDR-adjusted analysis (Supplementary Table S7).

### 3.5 Isolation and characterization of carbapenemase producers

Thirteen bacterial isolates were obtained from the biofilm and hospital wastewater samples (Supplementary Table S8). PCR screening for carbapenemase genes revealed the presence of *bla*_IMP_, *bla*_GES_, and *bla*_KPC_ among the isolates. Consistent with its higher transcriptomic expression in the biofilm, *bla*_IMP_ was found to be expressed in four biofilm isolates but in just one hospital-wastewater isolate. Whole-genome sequencing identified the biofilm isolates as *Pseudomonas alcaligenes* BFCARBA3 and *Aeromonas caviae* BFMDRP4, both carrying two *bla*_IMP-1_ genes, and *Citrobacter freundii* BFESBL3 and *P. aeruginosa* BFMDRP3, each carrying one *bla*_IMP-1_ gene (Table 1 and Supplementary Table S9). In addition to *bla*_IMP-1_, these four isolates harbored a diverse set of AMR genes, MRGs, BRGs, and HRGs. *bla*_IMP-1_ was found on the chromosome, IncFII plasmid, or on a plasmid of unknown incompatibility type. The *A. caviae* HWMDRP2 isolate obtained from hospital wastewater exhibited the same phylogenetic characteristics as *A. caviae* BFMDRP4 but lacked the 41,236-bp contig containing *bla*_IMP-1_. In antimicrobial susceptibility testing, *C. freundii* BFESBL3, *P. alcaligenes* BFCARBA3, and *P. aeruginosa* BFMDRP3 were resistant to carbapenems, whereas the *A. caviae* strains BFMDRP4 and HWMDRP2 were classified as susceptible; however, BFMDRP4 exhibited reduced susceptibility to meropenem (Table 2).

**Table 1:** Genomic features of IMP-1-producing isolates from *in situ* biofilm and hospital wastewater samples.

Strain	Species	MLST	Sequence component			Plasmidfinder	ARGs	MRGs	BRGs	HRGs
BFESBL3	*Citrobacter freundii*	No match	Contig1	4,859,215	Circular	-	*bla*_CMY-65_, *qnrB38*	*fieF*	-	-
			Contig2	234,476	Circular	IncFII(Yp)	*bla*_IMP-1_, *bla*_OXA-2_, *bla*_TEM-1_, *aac(6′)-Ib*, *aac(3)-Iid*, *aadA2*, *mph(A)*, *sul1*, *dfrA12*	*merA*, *merD*, *merE*, *merR*, *merT*	*qacEdelta1*	-
			Contig3	47,723	Circular	IncP6	*bla*_KPC-2_	-	-	-
			Contig4	45,455	Circular	IncX3	*qnrS1*	-	-	-
			Contig5	33,416	Circular	IncX6	-	-	-	-
BFCARBA3	*Pseudomonas* *alcaligenes*	Not applicable	Contig1	4,350,016	Liner	-	*bla*_IMP-1_ (2 copies), *bla*_OXA-932_, *aadA1* (2 copies), *aac(6′)-Ib*, *aac(6′)-Iic*, *aac(6′)-Il*, *sul1* (2 copies)	-	*qacEdelta1* (2 copies)	*psi-GI*, *kefB-GI*, *trxLHR*, *hdeD-GI*, *yfdX2*, *yfdX1*, *shsP*, *clpK* (2 copies)

BFMDRP3	*Pseudomonas aeruginosa*	ST235	Contig1	6,915,056	Circular	-	*bla*_IMP-1_, *bla*_OXA-1_, *bla*_OXA-488_, *bla*_PDC-35_, *aadA6*, *aac(6′)-Ib*, *aph(3′)-Iib*, *mexA*, *mexE*, *mexX*, *fosA*, *sul1* (2 copies), *catB7*	*merA*, *merD*, *merE*, *merP*, *merR*, *merT* (2 copies each)	*qacEdelta1* (2 copies)	-

BFMDRP4	*Aeromonas caviae*	ST1061	Contig1	4,630,085	Circular	-	*bla*_OXA-1160_, *bla*_MOX-23_, *aadA2*, *aph(3′)-Ia*, *dfrA12*, *mph(A)*, *sul1*, *tetE*	-	*qacEdelta1*	-
			Contig2	93,128	Circular	-	*bla*_IMP-1_	-	-	-
			Contig3	41,236	Circular	-	*bla*_IMP-1_	-	-	-
			Contig4	39,460	Circular	-	*bla*_GES-24_, *aac(6′)-Il*	-	-	-
HWMDRP2	*Aeromonas caviae*	ST1061	Contig1	4,628,224	Circular	-	*bla*_OXA-1160_, *bla*_MOX-23_, *aadA2*, *dfrA12*, *mph(A)*, *sul1*, *tetE*	-	*qacEdelta1*	-
			Contig2	93,128	Circular	-	*bla*_IMP-1_	-	-	-
			Contig3	39,460	Circular	-	*bla*_GES-24_, *aac(6′)-Il*	-	-	-

MLST: multi-locus sequence typing, ARGs: antimicrobial resistance genes, MRGs: metal resistance genes, BRGs: biocide resistance genes, HRGs: heat resistance genes.

**Table 2:** Antimicrobial susceptibility profiles of IMP-1-producing isolates from *in situ* biofilm and hospital wastewater samples.

Antibiotics	BFESBL3	BFCARBA3	BFMDRP3	BFMDRP4	HWMDRP2
Ampicillin	>16 (R)	ND	ND	>16	>16
Piperacillin	ND	16 (S)	>64 (R)	ND	ND
Ampicillin/sulbactam	>16/8 (R)	ND	ND	>16/8	>16/8
Piperacillin/tazobactam	>64/4 (R)	>64/4 (R)	>64/4 (R)	≤8/4 (S)	≤8/4 (S)
Ceftolozane/tazobactam	>4/4 (R)	ND	ND	>4/4	>4/4
Cefazolin	>16 (R)	ND	ND	>16	>16
Cefepime	>8 (R)	>16 (R)	>16 (R)	>8 (R)	4 (I)
Cefditoren	>2	ND	ND	>2	>2
Ceftazidime	>8 (R)	>16 (R)	>16 (R)	>8 (R)	>8 (R)
Ceftriaxone	>2 (R)	ND	ND	>2 (R)	>2 (R)
Cefpodoxime	>4 (R)	ND	ND	>4	>4
Cefpodoxime/clavulanic acid	>1	ND	ND	>1	>1
Cefozopran	ND	>16	>16	ND	ND
Cefmetazole	>32 (R)	ND	ND	>32	>32
Flomoxef	>32	ND	ND	>32	32
Latamoxef	>8	ND	ND	>8	>8
Aztreonam	>8 (R)	>16 (R)	4(S)	≤4 (S)	≤4 (S)
Imipenem	ND	>8 (R)	>8 (R)	ND	ND
Meropenem	>2 (R)	>8 (R)	>8 (R)	1 (S)	≤0.12 (S)
Doripenem	ND	>8	>8 (R)	ND	ND
Faropenem	>4	ND	ND	>4	>4
Colistin	≤1 (I)	>4	≤1 (I)	≤1	≤1
Gentamicin	>8 (R)	≤2 (S)	>8 (R)	≤4 (S)	≤4 (S)
Amikacin	≤16 (S)	≤8 (S)	16 (S)	≤16 (S)	≤16 (S)
Tobramycin	ND	≤2 (S)	>8 (R)	ND	ND
Minocycline	≤4 (S)	≤2 (S)	>8	≤4	≤4
Ciprofloxacin	ND	>2 (R)	>2 (R)	ND	ND
Levofloxacin	>1 (R)	2 (S)	>4 (R)	0.5 (S)	0.5 (S)
Trimethoprim/sulfamethoxazole	>2/38 (R)	>2/38 (R)	>2/38	>2/38 (R)	>2/38 (R)
Fosfomycin	≤4 (S)	ND	ND	>16	>16
Tigecycline	≤1	≤0.5	>2	≤1	≤1

S: susceptible, I: intermediate, R: resistance, ND: not determined.

### 3.6 Genetic context and phylogenetic relationship of *bla*_IMP-1_-harboring isolates

**Fig. 5:**
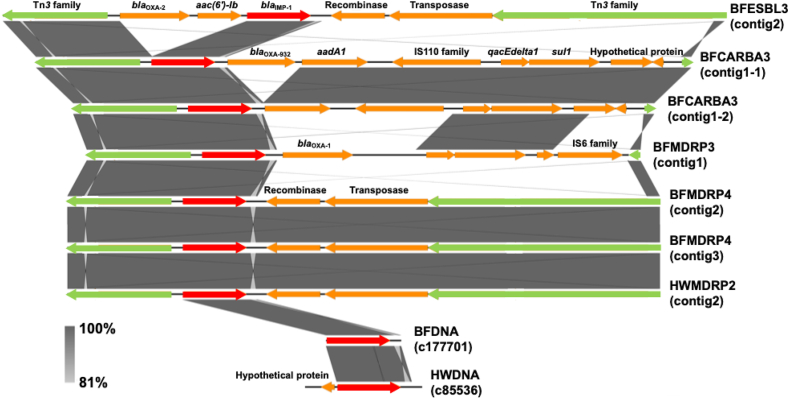
Linear comparison of genomic regions surrounding *bla*_IMP-1_. Gray shading indicates similarity between sequences. Arrows represent gene orientation: red, lime green, and orange represent *bla*_IMP-1_, the Tn*3* family sequence, and other genes, respectively. (For interpretation of the references to color in this figure legend, the reader is referred to the Web version of this article.)

**Fig. 6:**
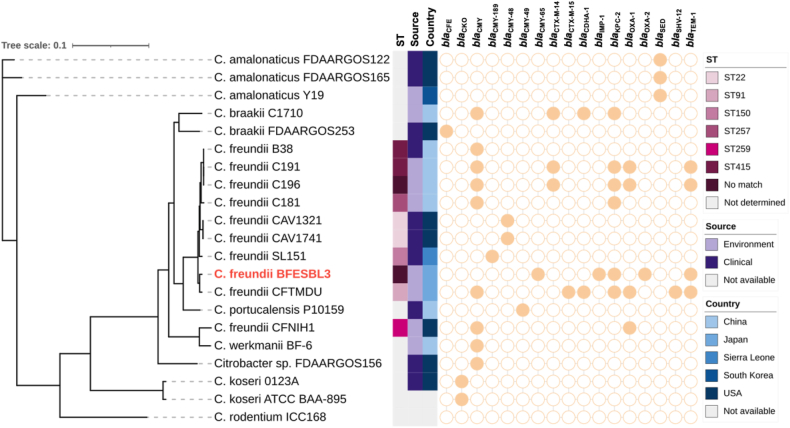
**Maximum-likelihood phylogenetic tree of *Citrobacter* species, including isolates from *in situ* biofilm sample and publicly available genomes.** The tree was constructed using core genome sequences and annotated with metadata including sequence type (ST), isolation source, country of origin, and β-lactamase gene profiles.

We compared the genomic contexts surrounding *bla*_IMP-1_ detected in the biofilm and hospital wastewater samples (Fig. 5). All *bla*_IMP-1_ genes isolated using culture-based methods were located adjacent to a Tn*3* family transposon. In contig1 of *P. alcaligenes* BFCARBA3, the surrounding structures of the two *bla*_IMP-1_ genes showed high similarity, except that one of them additionally carried *bla*_OXA-932_. The genes surrounding *bla*_IMP-1_ in BFMDRP4 and HWMDRP2 exhibited highly conserved structures. The genomic contexts surrounding *bla*_IMP-1_ were not identified in the metagenomic contigs BFDNA_c177701 and HWDNA_c85536. The phylogenetic analysis showed that *C. freundii* BFESBL3 was closely related to *C. freundii* strain CFTMDU (SAMN23449392), previously detected in an upstream hospital sink that carried a *bla*_KPC-2_-horboring IncP6 plasmid [52] (Fig. 6). Comparative analysis of carbapenemase gene profiles indicated that *C. freundii* BFESBL3 additionally harbored *bla*_IMP-1_.

## 4 Discussion

Hospital wastewater is a major hotspot for AMR that contributes to the accumulation of clinically important organisms [5]. The presence of antibiotic residues, including ampicillin, sulbactam, levofloxacin, sulfamethoxazole, and trimethoprim, previously detected in hospital wastewater from the same facility, could exert selective pressure promoting the emergence and persistence of AMR [55]. Biofilms play a central role in sustaining and spreading AMR because their extracellular matrix and structured architecture protect microbes from antibiotics and promote horizontal gene transfer [8,9]. Crystal-violet staining revealed structurally cohesive biofilm accumulation on the surface of the polystyrene slides placed in hospital wastewater. Additionally, fluorescence microscopy exhibited a highly heterogeneous 3D matrix composed of abundant nucleic acids, polysaccharides, and proteins, in line with observations that hospital-wastewater biofilms are EPS enriched in these components [15]. Nucleic acids form dense structural scaffolds that restrict antibiotic penetration and support the transfer of resistance determinants within the biofilm community [8,9]. Polysaccharides generate highly viscous diffusion barriers that adsorb or neutralize antimicrobial compounds, effectively lowering their local concentrations [56]. Structural and enzymatic proteins further stabilize the matrix and modulate stress responses, enhancing persister cell formation and occasionally binding or inactivating antibiotics [57]. Consistent with these EPS-mediated mechanisms, our culture-based assessment showed markedly higher contamination with ESBL-producing *E. coli* in biofilm than in wastewater, reflecting the enhanced AMR-accumulation characteristic of biofilm communities.

A recent metagenomic study demonstrated that hospital wastewater harbors diverse bacterial communities and a wide range of AMR genes [6]. Taxonomic differences between *in situ* biofilms and hospital wastewater reflect distinct ecological conditions in these environments. Hospital wastewater is a dynamic, planktonic matrix continuously influenced by fecal and clinical inputs, favoring gut-associated taxa such as *Bacteroidaceae* [58,59]. By contrast, biofilms represent stable, surface-associated habitats that preferentially select *Flavobacteriaceae*, a group widely associated with the formation of biofilm and the degradation of complex organic substrates in aquatic environments [60]. These ecological differences are also reflected in AMR gene profiles, with biofilms harboring a broader diversity of carbapenemase genes than hospital wastewater, consistent with stable reservoirs in biofilms and transient release in wastewater [9]. Virulence-associated genes encoding adhesins, toxins, and components of iron acquisition systems were predominantly detected in hospital wastewater, indicating direct inputs from infected or colonized patients and the enrichment of pathogenic gram-negative bacteria [59,61]. The scarcity of virulence genes in biofilms suggests dominance of environmentally adapted or opportunistic bacteria with limited pathogenic potential, despite their function as reservoirs of AMR genes.

Carbapenemase genes such as *bla*_KPC_, *bla*_IMP_, and *bla*_GES_ are commonly associated with Tn*3*-family composite transposons, which promote their duplication and horizontal dissemination across diverse chromosomal and plasmid backbones [62,63]. IS further enhances this process by introducing structural alterations or by disrupting local regulatory regions, and IS*4*, IS*6*, IS*30*, IS*1182*, and IS*1595* have been reported in association with *bla*_KPC_, *bla*_IMP_, *bla*_NDM_, and *bla*_OXA_ families [[64], [65], [66], [67]]. Differences in gene expression across environments reflect genomic context, as biofilm conditions preferentially activate resistance loci embedded in structurally dynamic regions [68,69]. Within biofilms, localized stress and close cell–cell proximity can promote DNA rearrangements around transposons and insertion sequences, increasing transcriptional responsiveness of the associated AMR genes. The strong transcriptional bias toward the *bla*_IMP_ family is consistent with this interpretation, as *bla*_IMP_ genes are typically embedded in Tn*3*-linked modules flanked by insertion sequences, which are highly responsive to structural rearrangement [62,63,66].

Culture-based isolation of carbapenemase-producing bacteria confirmed hospital-wastewater biofilms as reservoirs of clinically relevant AMR genes, with *bla*_IMP-1_ predominantly detected in biofilm. The detection of *bla*_IMP-1_ across phylogenetically distinct genera, including *C. freundii*, *P. aeruginosa*, *P. alcaligenes*, and *A. caviae*, highlights the broad host range of IMP-type carbapenemases and their capacity to circulate among environmental and opportunistic pathogens in hospital wastewater [3,70]. Whole-genome sequencing identified *bla*_IMP-1_ on chromosomes and on plasmids of multiple incompatibility types, pointing to its broad compatibility with diverse genomic backbones. A previous study has shown that IMP-type carbapenemases can exhibit variable carbapenem susceptibility patterns depending on the bacterial host and genetic background [71]. Similarly, OXA-type carbapenemases have been reported to exhibit altered resistance phenotypes in different genetic contexts [72]. Consistent with these observations, IMP-type carbapenemase-producing *Aeromonas* strains have been reported to remain susceptible to meropenem despite carrying carbapenemase genes [73]. Notably, two biofilm-derived isolates carried duplicate copies of *bla*_IMP-1_, and *A. caviae* BFMDRP4 was nearly genetically identical to *A. caviae* HWMDRP2 except for the acquisition of an additional *bla*_IMP-1_-encoding plasmid. Consistent with this difference, although both strains remained susceptible to meropenem, isolates with two *bla*_IMP-1_ exhibited lower susceptibility to meropenem than *A. caviae* HWMDRP2. Given that gene copy number is closely associated with expression level [74], the presence of isolates harboring multiple *bla*_IMP_ copies may have contributed to elevated overall *bla*_IMP_ activity within the *in situ* biofilm. Comparative analysis of *bla*_IMP-1_ genetic contexts revealed consistent localization adjacent to Tn*3* family elements, suggesting the potential involvement of plasmids and transposons in the dissemination of *bla*_IMP-1_ within biofilms. The predominance of IMP-type carbapenemases reflects the Japanese epidemiology, where *bla*_IMP_ is endemic in clinical and wastewater-associated isolates [75]. The IMP-type carbapenemase-producing *C. freundii* BFESBL3 strain also harbored a *bla*_KPC-2_-carrying IncP6 plasmid. The prior detection of *C. freundii* CFTMDU strain that was closely related at the whole-genome level and carried a *bla*_KPC-2_-harboring IncP6 plasmid in an upstream hospital sink suggests possible acquisition of *bla*_IMP-1_ within the hospital wastewater system [52]. These observations imply that the ecological success of IMP-type carbapenemases in biofilm-dominated environments may be associated with their genomic embedding within highly responsive mobile-element-associated regions that efficiently couple environmental stress to elevated activity at both molecular and phenotypic levels.

This study has some limitations. The single-facility design and limited number of samples restrict generalizability beyond this epidemiological setting, with the observed patterns potentially varying across healthcare institutions and sampling periods. Carbapenem resistance is shaped by distinct dissemination dynamics that differ from *bla*_IMP_-dominant contexts, potentially leading to alternative ecological outcomes [76]. Another limitation is the practical constraints of the approaches employed in this study. Isolation of carbapenemase producers relied on culture-based methods, which preferentially recover fast-growing, cultivable taxa and may underestimate AMR genes harbored by slow-growing or viable-but-nonculturable bacteria in wastewater and biofilm environments [77]. Additionally, metagenomic sequencing did not allow complete reconstruction of genomic regions surrounding *bla*_IMP-1_, limiting characterization of the local genetic context and comparability with culture-derived genomes. The co-occurrence analyses were based on co-detection patterns, and these analyses are useful for identifying potential associations, but do not necessarily indicate direct physical linkage, ecological interaction, or gene transfer. Furthermore, as the study was based on *in situ* environmental observations, the underlying gene transfer processes could not be directly evaluated. Future studies incorporating broader sampling alongside controlled experimental systems will be required to improve generalizability and clarify these processes.

This study demonstrates that *in situ* biofilms formed on plastic surfaces in hospital wastewater can act as important reservoirs of AMR. Biofilms harbored a broader diversity of carbapenemase determinants than planktonic wastewater and showed co-occurrence structures dominated by Tn*3* family transposon and IS linking AMR genes to diverse microbial hosts. The metatranscriptomic analysis revealed higher expression of *bla*_IMP_-family genes and mobile genetic elements in biofilms, indicating conditions potentially favorable for resistance persistence and horizontal dissemination under biofilm conditions. Culture-based isolation and whole-genome sequencing confirmed the widespread distribution of *bla*_IMP-1_ across multiple genera and genomic contexts associated with Tn*3* family transposons. These culture-based findings provided independent validation of the metagenomic and metatranscriptomic observations, particularly the presence of *bla*_IMP_-family genes in viable biofilm-associated bacteria. However, additional studies across multiple hospitals and sampling periods are needed to determine the broader generalizability of these patterns and to further clarify the implications of the observed co-occurrence relationships. Nonetheless, these findings underscore hospital-wastewater biofilms as critical interfaces for the persistence and dissemination of clinically relevant carbapenem resistance, highlighting the value of biofilm-focused approaches for early AMR detection and risk assessment within a One Health framework.

## CRediT authorship contribution statement

**Yusuke Ota:** Conceptualization, Formal analysis, Funding acquisition, Investigation, Project administration, Resources, Visualization, Writing – original draft. **Yoko Nukui:** Formal analysis, Writing – review & editing. **Yoshiaki Gu:** Formal analysis, Writing – review & editing. **Ryoichi Saito:** Conceptualization, Funding acquisition, Resources, Supervision, Writing – review & editing.

## Declaration of competing interest

The authors declare that they have no known competing financial interests or personal relationships that could have appeared to influence the work reported in this paper.

## Data Availability

Data will be made available on request.
