# Supplementary material for: Genomic insights into activated antimicrobial resistance of in situ hospital-wastewater biofilm
Source: Biofilm. 2026 Jun 23;12:100377. doi: 10.1016/j.bioflm.2026.100377 (PMC13320429; doi:10.1016/j.bioflm.2026.100377)
Supplement: Multimedia component 1 [file mmc1.docx]

**Supplementary Information**

**Genomic insights into activated antimicrobial resistance and mobile genetic elements of *in situ* hospital‑wastewater biofilm**

 **Supplementary Figure S1. Network analysis of plasmid-derived contigs, functional genes, and mobile genetic elements based on shotgun metagenomic data.**

Networks of plasmid-derived contigs and genes related to antimicrobial resistance (AMR), biocide and metal resistance, insertion sequences (ISs), and virulence are depicted for biofilm (**a**) and hospital wastewater (**b**) samples. Nodes represent individual sequences, and edges indicate co-occurrence within the same contig.
